# Suicidal behaviors among Moroccan school students: prevalence and association with socio-demographic characteristics and psychoactive substances use: a cross-sectional study

**DOI:** 10.1186/s12888-015-0680-x

**Published:** 2015-11-14

**Authors:** Btissame Zarrouq, B. Bendaou, S. Elkinany, I. Rammouz, R. Aalouane, B. Lyoussi, S. Khelafa, A. Bout, N. Berhili, H. Hlal, C. Nejjari, K. El Rhazi

**Affiliations:** Laboratory of Epidemiology, Clinical Research, and Health Community, Faculty of Medicine and Pharmacy, Sidi Mohammed Ben Abdallah University, Fez, Morocco; Laboratory of Physiology-Pharmacology and Environmental Health, Dhar El Mahraz Faculty of Sciences, Sidi Mohammed Ben Abdallah University, Fez, Morocco; Laboratory of Clinical Neurosciences, Faculty of Medicine and Pharmacy, Sidi Mohammed Ben Abdallah University, Fez, Morocco; Department of Psychiatry, Ibn Al Hassan Hospital, Hassan the 2nd University Hospital Center, Faculty of Medicine and Pharmacy, Sidi Mohammed Ben Abdallah University, Fez, Morocco; Department of English, Dhar El Mahraz Faculty of literature and human sciences, Sidi Mohammed Ben Abdallah University, Fez, Morocco; Department of Epidemiology and Public Health, Faculty of Medicine and pharmacy of Fez, Sidi Mohamed Ben Abdillah University, B.P 1893 Route Sidi Harazem, Km 2.2, Fez, Morocco

**Keywords:** Suicidal behavior, Suicide ideation, Suicide plan, Suicide attempts, Psychoactive substances use, Tobacco use, School students, Morocco

## Abstract

**Background:**

Suicidal behavior is a major cause of injury and death worldwide, especially among adolescents and young adults. Few studies have tackled this issue in the Arab world. The present study investigated the prevalence and the risk factors of suicidal behaviors among Moroccan school students.

**Methods:**

From April 2012 to November 2013, a cross-sectional study was conducted in the North-Centre region of Morocco among students in public secondary schools selected using stratified cluster random sampling. The data were collected via anonymous self-administered questionnaires. The Mini International Neuropsychiatric Interview was used in its Moroccan Colloquial Arabic version to assess suicidality according to the DSM-IV criteria.

**Results:**

A total of 3020 students (53 % boys) aged 11–23 years (average age = 16 ± 2.1 years) were included in the study. The prevalence of suicide ideation, suicide planning and suicide attempts during the last month were 15.7, 6.3, and 6.5 % respectively.

Univariate analyses demonstrated that suicidal behaviors followed different epidemiological patterns. According to the multivariate analyses, the risk factors for all suicidal behaviors among Moroccan school students were the female gender, middle school level, urban locations, low family income, parents’ divorce, tobacco consumption and psychoactive substances (alcohol and cannabis) use.

**Conclusions:**

The intervention of preventive programs has become an emergency to overcome the issue of suicidality in Morocco. Further researches on adolescents’ suicidal behaviors are suggested to update temporal data and assess the effectiveness of potential interventions.

**Electronic supplementary material:**

The online version of this article (doi:10.1186/s12888-015-0680-x) contains supplementary material, which is available to authorized users.

## Background

Suicidal behavior comprises a wide range of thoughts and acts intended to end one’s life, such as suicidal ideation, suicide planning, and suicide attempts [1]. Suicidal ideation refers to thoughts of engaging in behavior intended to end one’s life, whereas suicide planning is defined as the formulation of a specific method through which one intends to die. Finally, attempted suicide is understood as an engagement in potentially self-injurious behavior in which there is at least some intent to die [2, 3].

Suicidal behaviors are among the main causes of death worldwide, especially among adolescents and young adults [4, 5]. It is estimated that for every suicide that occurs worldwide, there are up to 20 suicide attempts [6]. According to the World Health Organization report (2014), the global median rate of suicide was 6.55 per 100 000 people, compared to 4.90 in the countries of the Eastern Mediterranean Region [6].

Based on the previous findings, suicide may not seem as one of the most urgent health problems in this region. In fact, suicides simply die uncounted in countries without reliable registration of deaths [6]. Moreover, in some countries of the Eastern Mediterranean Region, such as Morocco, there is no national register for suicide. The only reported suicide deaths are the ones which occur inside Moroccan hospitals. Other suicide deaths may often be misclassified as an accident or another cause of death to avoid familial, religious, and social stigmatizations. Given the fact that suicide and attempted suicide are considered disgraceful acts prohibited by religion, condemned by society, and hampered by legal consequences, suicidality remains a taboo in this region of the world [7].

As a matter of fact, the epidemiological studies investigating the prevalence of suicidal behaviors and their correlates are scarce. In Morocco; for instance, only one survey was conducted among the general population, based on face-to-face household interviews. The results showed that the prevalence of suicide attempts and suicidal ideation during one month were 2.1 and 6.3 %, respectively [8]. The Global School Health Survey (GSHS) conducted by the World Health Organization, in collaboration with the US Centers for Disease Control and Prevention, provided an important opportunity to rectify the lack of data on youth suicidal behavior. The GSHS conducted in 2010 among Moroccan school students aged 13–15, showed that the past 12 month prevalence of suicide ideation ranged from 13.3 to 18.4 %; while, suicide attempts reached 13.3 % [9].

Concerning the risk factors of youth suicidal behaviors, the most frequently reported ones were mental health problems, alcohol as well as psychoactive substances (PASs) use, and poor familial or social bonds [10–12].

Obviously, the data on the issue of Moroccan youth suicidality are rare. Henceforth, the presented study aimed at determining the prevalence and the correlates of suicidal behaviors among school students in the North-Centre of Morocco.

## Methods

### Research design

This cross-sectional study was conducted from April 2012 to November 2013 (Additional file 1). The project was approved by the Ethics Committee of the Faculty of Medicine and pharmacy of Sidi Mohammed Ben Abdellah University. The participants were students from middle and high public schools in the North Central Region of Morocco (namely regions of Fez, Boulemane, Taza, Tawnat, and Hoceima). Based on the lists of schools in this region, which were obtained from the Statistical Office of National Education Ministry, schools were split into seven geographic regions, and classified into two strata: urban and rural. The stratified random sampling was carried out according to the type of schools (middle or high school) using the proportional allocation technique, that is to divide the sample in each stratum (urban and rural) based on the proportionality of middle and high schools depending on the total number of schools. Eventually, 44 middle schools and 24 high schools were randomly selected. Indeed, all the students from the selected classes were included in the study. The questionnaires (Additional file 2) were administered upon receiving school-directors permissions and verbal informed consents from all participants. Concerning the students under the age of 16, verbal informed consents from the parents were also obtained. The process of contacting parents was facilitated through parents’ associations.

Trained resident doctors on psychiatry distributed the questionnaires in the classrooms during a regular class period. The questionnaires’ instructions stated that participation was voluntary and that responses were anonymous and confidential.

### Assessment

#### Socio-demographic characteristics

A questionnaire was used to collect demographic information such as age, gender, educational level, family income, parents’ marital status and their educational levels.

### Suicidal behavior

To asses suicidal behavior according to the DSM-IV criteria [13], The Mini International Neuropsychiatric Interview (MINI) [14], was used in its Moroccan Colloquial Arabic version [15]. THE MINI suicidality module involved 6 items; each one consisted of a dichotomous response type (Yes/No), and all questions were relevant to suicidal behaviors including death wish, self-harm wish, suicide ideation, suicide plan, suicide attempt in the past month, and lifetime suicide attempts.

### Psychoactive substances use

As stated in the report of the Mediterranean School Project on Alcohol and Other Drugs, the most used substance among Moroccan school students was tobacco, followed by alcohol, then cannabis [16]. Thus, each respondent in this study was asked on his or her tobacco, alcohol, and cannabis use during their lifetime. The types of smoking habits were determined according to the International Union against Tuberculosis and Lung Diseases guide [17]. Moreover, a lifetime psychoactive substances use (alcohol and cannabis use) was considered in case of the consumption of these psychoactive substances at least once in the lifetime.

### Statistical analyses

The data entry stage started immediately after data collection. Data were entered into MS Windows Excel in the form of codes, and transferred to the Statistical Package for Social Sciences (SPSS) software version 20 (SPSS Inc., Chicago, IL, USA). Data analysis involved descriptive as well as inferential statistics. A simple descriptive analysis was done for the variables of interest. Prevalence with 95 % confidence intervals (95 % CI) was also estimated. Odds ratios, along with 95 % confidence intervals, were calculated. Differences in proportions were assessed by the Chi-square test. *P* values of <0.05 were considered as statistically significant.

## Results

### Socio-demographic characteristics

Out of 3170 students who participated in the study, 3020 completed the questionnaires (response rate = 95.2 %). There were more males (53 %) than females (47 %), and the average age was 16 years (SD = 2.1 years). Other socio-demographic characteristics of the sample were displayed in Table 1.

**Table 1:** Sample characteristics (*N* = 3020)

Characteristics	Number	Percent
Gender		
Boys	1602	53
Girls	1418	47
Age group		
11–14	795	26.4
15–18	1857	61.7
19–23	357	11.9
School level		
Middle school	1657	54.9
High school	1363	45.1
School location		
Urban	2273	75.3
Rural	774	24.7
Parents’ marital status		
Married	2684	89.3
Divorced	109	3.7
Widowed	145	4.9
Separated	65	2.1
Father education		
Illiterate	1065	35.9
Primary	806	27.2
Secondary	767	25.9
University and above	326	11
Mother education		
Illiterate	1813	60.6
Primary	514	17.2
Secondary	517	17.3
University and above	149	5
Father employment		
Employed	2490	88.4
Unemployed	82	2.9
Retired	245	8.7
Mother employment		
Employed	281	9.5
Unemployed	2688	90.5
Family income		
≤ 300 £	498	16.8
301–1000 £	2280	76.9
≥ 1000 £	187	6.3
Smoking		
Non smokers	2532	83.9
Current smokers	275	9.1
Ex-smokers	210	7.0
Lifetime PASs use		
No	2713	90.6
Yes	280	9.4

### Psychoactive substances use

As showed in Table 1, the prevalence of current smokers was 9.1 % (95 % CI: 8.1 %–10.2 %). Male students were more likely to smoke cigarettes than females (15 % vs. 2.5 %; *p* < 0.001). As for psychoactive substances consumption, the overall lifetime prevalence was 9.4 % (95 % CI: 8.35 %–10.47 %). Likewise, the rate of psychoactive substances use was higher among boys than girls (15.74 % vs. 2.13 %; *p* < 0.001). Cannabis recorded the highest lifetime prevalence with 8.08 % (13.53 among boys vs. 1.92 among girls), followed by alcohol 4.31 % (7.49 among boys vs. 0.71 among girls).

### Prevalence of suicidal behaviors

The most prevalent suicidal behavior during the last month among the studied sample was death wish (26.6 %), followed by suicide ideation (15.7 %), and self-harm wish (14.7 %). Concerning the suicide attempts which occurred in the last month (6.5 %), the majority were planned (6.3 %). Life time suicide attempts on the other hand reached 10.5 % (Table 2).

**Table 2:** Prevalence of suicidal behaviors among Moroccan school students

Suicidal behaviors	Number	Percent	95 % CI
In the past month, did you			
Think that you would be better off dead or wish you were dead? (Yes)	768	26.6	25–28.2
Want to harm yourself? (Yes)	426	14.7	13.5–16.1
Think about suicide? (Yes)	453	15.7	14.4–17.1
Have a suicide plan? (Yes)	182	6.3	5.4–7.2
Attempt suicide? (Yes)	187	6.5	5.6–7.4
In your life			
Did you ever make a suicide attempt? (Yes)	304	10.5	9.4–11.7

### Correlations of socio-demographic characteristics and suicidal behaviors

The female gender was the only socio-demographic variable which displayed significant association with all suicidal behaviors. For the other socio-demographic characteristics, suicidal behaviors followed various epidemiological patterns as illustrated in Table 3.

**Table 3:** Socio-demographic characteristics and their correlations with suicidal behaviors among Moroccan school students

	Past month death wish		Past month self-harm wish		Past month suicide ideation		Past month suicide plan		Past month suicide attempt		Lifetime suicide attempts	
	(%)	*P* value	(%)	*P* value	(%)	*P* value	(%)	*P* value	(%)	*P* value	(%)	*P* value
Gender		<0.0001		<0.001		<0.0001		0.004		0.02		<0.0001
Boys	17.5		12.6		10.9		5.1		5.5		7.8	
Girls	36.8		17.1		20.9		7.6		7.4		13.5	
Age (years)		0.003		0.03		0.04		0.3		0.7		0.1
11–14	22.3		12		12.8		6.5		6.7		10.8	
15–18	28.8		15.9		16.6		6.5		6.5		10.9	
19–23	25.4		14.7		17.1		4.4		5.6		7.6	
School level		0.009		0.2		0.3		<0.0001		<0.0001		0.001
Middle school	24.8		15.2		15.4		7.7		7.9		12.2	
High school	28.8		14.3		16.1		4.6		4.7		8.5	
School location		<0.0001		0.01		<0.0001		0.06		0.1		0.03
Rural	20.4		12.3		11.1		5		5.6		8.7	
Urban	28.7		15.6		17.3		6.7		6.8		11.2	
Parents’ marital status		<0.0001		0.007		0.001		0.01		<0.0001		0.1
Married	25.4		14		14.9		5.8		5.9		10.2	
Divorced	37.7		22.6		24.8		13.2		16		15.1	
Widowed	33.3		17.4		15.9		8		8.7		13.1	
Separated	45.2		24.2		30.6		9.7		8.1		9.7	
Father education		0.016		0.004		0.01		0.1		0.006		0.4
Illiterate	25.6		13.7		14.2		5.8		6.4		9.8	
Primary	27		15.4		15.9		5.7		5.9		11.4	
Secondary	30		17.5		19.1		8		8.5		11.4	
University and above	20.8		9.3		12.2		4.5		2.9		8.6	
Mother education		0.07		0.1		0.2		0.9		0.03		0.07
Illiterate	26.2		14.9		15		6		6.7		10.5	
Primeray	24.5		12.7		14.5		6.2		4		8.4	
Secondary	31		16.5		18.5		6.8		7.8		13.1	
University and above	23.4		9.9		14.2		5.7		3.5		7.7	
Father employment		0.3		0.06		0.2		0.1		0.2		0.06
Unemployed	33.3		22.2		22.2		11.1		8.6		12.3	
Employed	26.2		14.2		15.5		6.1		6.5		10.9	
Retired	25.3		17.2		14.6		5.2		4.3		6	
Mother employment		0.07		0.1		0.01		0.1		0.07		0.09
Employed	30.5		16.8		15		8.2		8.6		13.1	
Unemployed	26.2		14.3		20.5		6		6.1		10.2	
Family income		<0.0001		<0.0001		<0.0001		0.08		0.001		0.01
≤ 300 £	34.1		22.8		23.1		8.4		9.6		13.9	
301–1000 £	25.1		12.4		14.1		5.7		5.4		9.5	
≥ 1000 £	25		18.1		13.1		5.7		9.1		12.4	

### Suicidal behaviors in smokers and psychoactive substances (PASs) users

The prevalence of suicide ideation, planning and attempts during the last month among cigarettes smokers was 25.1, 10.9, and 12.2 %, respectively. Among PASs users, the prevalence was 26.7 , 14.9, and 16.5 %, respectively. Upon comparing suicidality based on tobacco and PASs use status, all suicidal behaviors were highly detected among smokers and users of PASs conversely to non-smokers and non-users, as shown in Table 4.

**Table 4:** Comparison of suicidal behaviors according to tobacco, and psychoactive substances use status

	Tobacco		*P*-value	Psychoactive substances		*P*-value
	Smokers (%) [95 % CI]	Non-smokers (%) [95 % CI]		Users (%) [95 % CI]	Non-users (%) [95 % CI]	
Death wish	34.1 [29.8–38.7]	25.2 [23.5–27]	<0.001	36.8 [30.9–43.1]	25.5 [23.8–27.2]	<0.001
Self-harm wish	27 [23–31.4]	12.4 [11.1–13.8]	<0.001	31.7 [26.1–37.8]	13.1 [11.8–14.4]	<0.001
Suicide ideation	25.1 [21.2–29.4]	14 [12.6–15.4]	<0.001	26.7 [21.4–32.6]	14.7 [13.3–16.1]	<0.001
Suicide plan	10.9 [8.2–14.2]	5.4 [4.6–6.4]	<0.001	14.9 [10.8–19.9]	5.5 [4.7–6.5]	<0.001
Suicide attempt in the past month	12.2 [9.4–15.6]	5.4 [4.5–6.4]	<0.001	16.5 [12.1–21.6]	5.5 [4.7–6.5]	<0.001
Lifetime suicide attempts	16.2 [13–20]	9.5 [8.3–10.7]	<0.001	19.6 [14.9–25.1]	9.6 [8.5–10.9]	<0.001

### Multivariate analysis

The binary logistic regression confirmed that suicidal behaviors did not follow the same epidemiological patterns. On the whole, socio-demographic risk factors for all suicidal behaviors among Moroccan school students were: the female gender, low family income, middle school level, urban locations, parents’ divorce. Parents’ educational level and parents’ employment however exhibited statistically non-significant association.

For the association of suicidal behaviors with tobacco and PASs use, the results showed that current smoking (OR = 2.7, *p* < 0.0001) was more associated with suicidal ideation than PASs use (OR = 1.8, *p* = 0.004); while, lifetime alcohol use and cannabis consumption (OR = 3.6, *p* < 0.0001) were more associated with past month suicide attempts than tobacco use (OR = 1.9, *p* = 0.007).

## Discussion

The present study aimed at determining the prevalence and the risk factors of suicidal behaviors mainly suicide ideation, suicide planning, and suicide attempts among Moroccan school students. Regarding suicide ideation, the prevalence was 15 % in subjects aged 13 to 15 years, which was almost identical to the national prevalence (15.7 %) reported by the 2010 Morocco GSHS [9]. The reported prevalence in Morocco was indeed similar to the GSHS results in other Arab countries, (15 %) in Lebanon in 2011, and (15.5 %) in United Arab Emirates in 2010; while, it was lower than the GSHS findings in Tunisia in 2008 (19.8 %) and in Mauritania in 2010 (31.1 %) [18]. Contrariwise, the overall prevalence of suicide ideation among Moroccan school students was among the lowest in comparison with other African countries (Benin, Ghana, Kenya, Zambia), some countries in the Americas (Chile, Guyana, and Santa Lucia), some European and Asiatic countries (Austria, Turkey, China), in which suicidal ideation was found to be very common among youth (up to 44 % among high school students) [18–20].

Concerning suicide attempts, it was demonstrated that the lifetime prevalence of these attempts among Moroccan school students (10.5 %) was seemingly equivalent to the average global rate (9.7 %) as reported in a systematic review from the international literature on the prevalence of suicidal phenomena among 513,188 adolescents in 2005 [21]. Nevertheless, the self-reported lifetime prevalence of suicide attempts in the present study (10.5 %) proved high in comparison with American and European countries. According to a recent survey conducted among 6483 American adolescents in 2013, the estimated lifetime prevalence of suicide attempts was 4.1 % [22]. Indeed, the Saving and Empowering Young Lives in Europe (SEYLE) project (2014), conducted across 11 European countries, revealed that 4.2 % adolescents attempted suicide during their lifetime [23].

The previous findings are surprising, in the sense that the prevalence of suicide ideation was low; while, the prevalence of suicide attempts proved high compared with western countries.

In essence, any comparison with western countries should be interpreted with caution due to the methodological differences present across suicide studies; therefore, it is difficult to make generalizations about the prevalence of suicidal behaviors.

To explain the previous contrast in the light of Moroccan culture specificities, suicide ideation low prevalence can be relegated to the religious and cultural prohibition of suicide, or even suicide thoughts. To illustrate, Baasher argued that Islam was behind the low rates of suicidal behaviors recorded in Muslim communities [24]. In general terms, an expanding body of literature has thoroughly examined religiosity as a potential protective factor against the development and the persistence of mental disorders and risk behaviors among youth [25, 26].

Despite the fact that all participants in this study adhere to Islam, the lifetime prevalence of suicide attempts hints on youth non-conformist or deviant religious attitudes. Among the limitations of this study is unfortunately the absence of any question about religiosity degrees since there is no validated scale in Morocco developed to assess such a variable.

The high prevalence of suicide attempts can also be interpreted in relation to the ongoing economic and social changes occurring in the Moroccan society, which have created a growing rift between younger and older generations, and led to intensifying conflicts between Moroccan youth and the conservative society. Likewise, adults still perceive youth problems as superficial. Thus, suicide attempts could be a call for communication in a society that pays no heed to youth dilemmas. For this reason, medically non-serious suicide attempts are more common than medically serious ones. The present study, be it a community-based survey, tends to represent all suicidal attempts regardless of their severity degree. Generally speaking, medically non-serious suicide attempts are more reported than medically serious ones. For example, although 8.0 % of United States students (grades 9 to 12) reported suicide attempts in the past year, the proportion reporting medically serious attempts- treated by a doctor or a nurse- was only 2.7 % [27].

The most important characteristic of attempted and completed suicide is the extent to which the act was planned. In this study, 6.3 % of students reported having made a suicide plan in the past month. For unplanned suicide attempts, the prevalence among Moroccan school students was unknown. Internationally, several studies revealed that the prevalence of unplanned or impulsive acts of suicide estimates has exceeded 50 % among adolescents [28].

On the account of these alarming results, attention shifted towards the study of suicide risk factors. The obtained results showed that all suicidal behaviors were more common among girls than boys. Such findings were consistent with those reported internationally. Lewinsohn et al. found that the prevalence rates of suicide attempts are two to three times higher in females than in males [29]. Rezaeian using data from the World Health Organization Global Burden of Disease Project, reported that in the Eastern Mediterranean Region, the rates of suicide were higher in females than males in age groups 5–14 and 15–29 years [30]. Even for females aged 18–65, Weissman et al. noted that compared with males, females had higher rates of suicidal ideation and suicide attempts in nine countries (The United States, Canada, Puerto Rico, France, West Germany, Lebanon, Taiwan, Korea and New Zealand) [31]. Focusing on suicide attempts, the expression is mostly associated with gender-specific norms. Males for instance are more inclined towards risk-taking behaviors, and PASs use. Eventually, their suicide attempts are more likely to result in fatalities as they employ violent means and irreversible methods. Concerning females, gender specific norms, in conjunction with low social status and the lack of economic and personal power can result in more suicide ideation, rumination, depression, and nonfatal suicide attempts [32, 33]. Suicide rates as matter of fact are typically higher in males than females, and the opposite for suicide attempts. This gender paradox was clarified in regards to genetic vulnerabilities and gender differences in early adverse environments, neurodevelopment, mental Disorder, social expectations and their complex interconnections [34].

In this study, an association was detected between tobacco consumption, PASs use, and suicidal behaviors. Current smoking was more associated with suicidal ideation and PASs consumption with suicide attempts. Such findings were consistent with other surveys depicting the consumption of tobacco and PASs as strong risk factors of suicidal behaviors [12, 35, 36]. Evren et al. and Juan et al. indicated a direct association between suicidal ideation and current smoking of cigarettes [37, 38]. Likewise, drinking alcohol was directly associated with suicidal attempts. Certain characteristics of alcohol use appeared to be predictive of attempted suicide, such as high alcohol consumption, and the uptake of strong alcoholic drinks [39, 40]. Moreover, alcohol use could facilitate suicide attempts; it was observed that many subjects use some substance mostly alcohol before committing suicide [41]. Equally important, most longitudinal studies proved that early adolescent cannabis use increased the risk of later depression and suicidal behaviors [42–44]. In fact, the relationship between suicidal behaviors, tobacco and PASs is complicated. The association has been well established, yet causal links remain unclear because suicidal behaviors, tobacco and PASs use are multiply determined. To illustrate, the use of certain PASs alongside with suicidal behaviors are related with strong feelings of escapism [45, 46].

Similarly to our findings, previous studies on the potential risk factors of suicidal behaviors revealed that socioeconomic deprivation, poverty and stress or worry about the economic situation of the family were relevant correlates to suicidal behaviors among adolescents [47, 48]. The association between parents’ cohabitation status and suicidal behaviors has already been investigated in many studies, and showed an indirect association between these two factors [39]. Family harmony on the other hand was found to have a stronger effect on reducing suicidal behaviors risk.

At last, the results of the present study should be considered in the light of several limitations. The study was based on a cross sectional sample, which indicated that the causal relations could not be determined. Indeed, despite of employing validated instruments to measure suicidality, the survey used self-reported data, which may reduce objectivity and introduce the possibility of misreporting. Furthermore, the sample consisting of school students cannot be considered as a representative sample of all Moroccan youth, because the study did not include unschooled teens. Finally, the study lacked data on characteristics such as mental disorders, parents’ psychopathology, sexual abuse, and adverse childhood experiences; hence, the impact of these characteristics on suicidal behaviors could not be studied. Further research needs to be conducted in this regard.

Concerning the strengths of the present study, it included a large representative sample. It was the first study in kind to be performed among middle and high school students in the North Centre region of Morocco with the aim of investigating the prevalence and exploring the risk factors of suicidal behaviors.

## Conclusions

Beyond these descriptive epidemiological results, an urgent intervention should be sought in favor of developing an integrated management approach to solve youth suicide problems. Since the findings of the current study have accentuated the pitfalls of youth mental health policies in Morocco, which do not consider the variations of suicidal behaviors depending on gender, PASs use status, and differences in socio-demographic characteristics. Even though suicidal ideation and suicidal attempts can be predictors of later suicide, the prediction of suicide remains very complex. There is therefore a need for the development of special prevention and intervention programs focusing on suicidal behaviors.

These prevention efforts need to be supported by parents and teachers who are supposed to pay more attention to adolescents’ risky behaviors, bearing in mind that the latters can be a “cry for help”. Further surveys on the suicidal behaviors of Moroccan adolescents are required to get temporal data and assess the effectiveness of future policies and interventions.

## Additional files

Additional file 1:
**STROBE checklist for cross sectional studies.** (DOC 88 kb) Additional file 2:
**English version of the questionnaire.** (DOCX 26 kb)

## Abbreviations

GSHS: Global School Health Survey
PASs: psychoactive substances
