# Supplementary material for: Suicidal behaviors among Moroccan school students: prevalence and association with socio-demographic characteristics and psychoactive substances use: a cross-sectional study
Source: BMC Psychiatry. 2015 Nov 14;15:284. doi: 10.1186/s12888-015-0680-x (PMC4647598; doi:10.1186/s12888-015-0680-x)
Supplement: Additional file 2: — English version of the questionnaire. (DOCX 26 kb) [file 12888_2015_680_MOESM2_ESM.docx]

Questionnaire ID……………

**Sidi Mohammed Ben Abdallah University**

**Dhar El Mahraz Faculty of Sciences**

**Ibn Al Hassan Hospital**

**Hassan the 2^nd^ University Hospital Center**

**Faculty of Medicine and Pharmacy, Fez, Morocco**

***A Questionnaire on Suicidal Behaviors and Substances Use***

| **Read this first please!**  Dear Student,  This questionnaire is a part of a study conducted on suicidal behaviors and substance use among Moroccan school students. It will be answered by more than 3000 students in the regions of Fez, Boulemane, Taza, Tawnat, and Hoceima.  Please, do not write your name on this questionnaire. Hence, nobody will be able to identify who has completed this particular form.  Please, answer as thoughtfully and as honestly as possible all the questions. This is not a test; therefore, there are no ‘right’ or ‘wrong’ answers.  Your class has been randomly selected to take part in this study. Completing the survey is voluntary. Your grade or mark in this class will not be affected whether or not you answer the questions. If you do not want to answer a particular question, just leave it blank.  Please, mark the appropriate answer to each question by making an "X" in the box. If you have a question, please raise your hand and your survey investigator will assist you.  **Thank you in advance for your participation! Please begin.** |
| --- |

**THE NEXT TEN QUESTIONS ASK FOR SOME BACKGROUND INFORMATION ABOUT YOURSELF.**

1. What is your sex?

❒ Male

❒ Female

1. How old are you? …….. Years
2. In what school level are you?

❒ Middle school

❒ High school

1. What is your school district?

❒ Urban

❒ Rural

1. As of today, what is the marital status of your legal Parents?

❒ Married

❒ Divorced

❒ Widowed

❒ Separated

#### What is your father’s highest completed level of education?

❒ No formal education

❒ Primary (1-6)

❒ Secondary (7-12)

❒ University and above

❒ I don’t know

1. What is your mother’s highest completed level of education?

❒ No formal education

❒ Primary (1-6)

❒ Secondary (7-12)

❒ University and above

❒ I don’t know

1. What does your father do?

❒ Employed

❒ Unemployed

❒ Retired

1. What does your mother do?

❒ Employed

❒ Unemployed

❒ Retired

1. What is your family monthly income?

❒ ≤ 3000 DH (300 £)

❒ 3100 – 10000 DH (301 – 1000 £)

❒ ≥ 10000 DH (1000 £)

**THE NEXT FIVE QUESTIONS ASK ABOUT YOUR USE OF CIGARETTES**

11- Have you ever tried cigarette smoking, even one or two puffs?

❒ Yes

❒ No

1. How many cigarettes have you smoked during your lifetime?

❒ 0 Cigarettes

❒ Less than 100 cigarettes

❒ More than 100 cigarettes

1. During the past 30 days, how many days did you smoke cigarettes?

❒ 0 days

❒ 1 or 2 days

❒ 3 to 5 days

❒ 6 to 9 days

❒ 10 to 19 days

❒ 20 to 29 days

❒ All 30 days

1. During the past year, have you ever tried to stop smoking cigarettes?

❒ I have not smoked cigarettes during the past year

❒ Yes, I have tried to stop smoking during the past year

❒ No, I have not tried to stop smoking during the past year

1. How long ago did you stop smoking?

❒ I have never smoked cigarettes

❒ I have not stopped smoking

❒ I have stopped smoking 1-3 months ago

❒ I have stopped smoking 4-11 months ago or longer

**THE NEXT TWO QUESTIONS ASK ABOUT THE USE OF ALCOHOL (BEER, WINE, SPIRITS)**

1. Have you ever tried alcohol drinking, even few sips?

❒ Yes

❒ No

1. On how many occasions (if any) have you had any alcoholic beverage to drink in your lifetime?

❒ 0

❒ 1–5

❒ 6–9

❒ 10–19

❒ 20 or more

**THE NEXT TWO QUESTIONS ASK ABOUT THE USE OF HASHISH (CANNABIS)**

1. Have you ever used Hashish?

❒ Yes

❒ No

1. On how many occasions (if any), have you used Hashish in your lifetime?

❒ 0

❒ 1–5

❒ 6–9

❒ 10–19

❒ 20 or more

**THE NEXT SIX QUESTIONS ASK ABOUT SUICIDAL BEHAVIORS**

***In the past month, did you:***

1. Think that you would be better off dead or wish you were dead?

❒ Yes

❒ No

1. Want to harm yourself?

❒ Yes

❒ No

1. Think about suicide?

❒ Yes

❒ No

1. Have a suicide plan?

❒ Yes

❒ No

1. Attempt suicide?

❒ Yes

❒ No

***In your life***

1. Did you ever make a suicide attempt?

❒ Yes

❒ No

**Thank you for your collaboration**
